# Investigation of Fatigue Load Spectrum Enhancement via Equivalent Plastic Zone

**DOI:** 10.3390/ma18215026

**Published:** 2025-11-04

**Authors:** Lindong Chai, Penghui Wang, Yifu Wang, Yihai He, Wei Zhang

**Affiliations:** 1 School of Reliability and Systems Engineering, Beihang University, Beijing 100191, China; chailindong@buaa.edu.cn (L.C.); adewang@buaa.edu.cn (Y.W.); hyh@buaa.edu.cn (Y.H.); 2 State Key Laboratory of Advanced Forming Technology & Equipment, Beijing 100083, China; 3 Beijing Institute of Structure & Environment Engineering, Beijing 100076, China; wph820831@163.com; 4 School of Astronautics, Northwestern Polytechnical University, Xi’an 710072, China

**Keywords:** accelerated life testing, load spectrum enhancement, fatigue life prediction, variable amplitude loading, equivalent plastic zone

## Abstract

Load spectrum enhancement is a pivotal accelerated fatigue testing methodology employed to substantially reduce test duration and associated costs. This technique operates by strategically elevating load amplitudes while ensuring the preservation of the original failure mechanism. In this study, a novel fatigue life prediction model for variable amplitude loading is developed by integrating the theories of Equivalent Initial Flaw Size (EIFS) and the Equivalent Plastic Zone (EPZ). This integrated approach explicitly accounts for both the small crack effect and load interaction effects, which are critical yet often oversimplified aspects of fatigue damage accumulation. The model is subsequently applied to quantitatively establish the relationship between the Load Enhancement Factor (LEF) and the test time or compression ratio. Finally, fatigue tests on typical 2A14 aluminum alloy structures under variable amplitude loading are conducted to validate the proposed model. The results demonstrate a significant life reduction with increasing LEF, achieving a remarkable test time reduction of over 50% at an LEF of 1.2. All experimental data fall within a scatter band of three, relative to the model prediction. Additionally, the predicted mean compression ratio exhibits approximate agreement with the experimental data, with errors within an acceptable range. This work provides a physically grounded and practically validated framework for implementing efficient and reliable load spectrum enhancement.

## 1. Introduction

The increasing design lifetimes of aircraft have led to significantly longer ground fatigue tests [1]. To effectively reduce test durations and associated costs, meticulous processing of the load spectrum is essential. Commonly used accelerated techniques include small load truncation [2,3], load equivalence [4], and load enhancement [5]. Among these methods, load enhancement is widely adopted due to its operational convenience in load spectrum processing. This method is usually achieved by proportionally increasing the original load spectrum while ensuring that the failure mechanism remains unchanged. The scaling factor applied in this process is referred to as the load enhancement factor (LEF).

Establishing the relationship between the accelerated factor (or test time compression ratio) and the LEF is central to load enhancement. Extensive research has been devoted to this topic. For instance, fatigue tests on Airbus A320 and Boeing 777 empennage structures employed a LEF of 1.25 [6]. The LEF of fatigue tests on the composite aircraft structures of the Starship was taken as 1.15 by massive experiments [7]. Dong et al. [8] derived the relationship between fatigue life and LEF using the structural detail fatigue rating (DFR) and damage accumulation theory. Dang et al. [9] further refined Dong’s method by ensuring the invariance of Ground–Air–Ground load. Although these methods are convenient for engineering applications, they are largely phenomenological or empirically based, lacking support from interpretation of the fatigue mechanism. To address this limitation, some researchers have attempted to establish this relationship based on linear elastic fracture mechanics (LEFM). Zhang et al. [10] derived an exponential relationship between accelerated and original crack growth life using the Paris model. Zhang et al. [11] proposed a formula relating crack propagation life to the LEF considering the crack closure, which is particularly effective for constant amplitude (CA) loading spectra. For variable amplitude (VA) loading, where load interaction effects are significant, Zhang et al. [12] modified Walker’s model and derived the relationship between accelerated factor with the LEF. Generally, these models provide valuable insights but share a common limitation: they predominantly focus on long crack growth behavior, thereby neglecting the critical influence of the small crack growth phase on the total fatigue life. This oversight can lead to non-conservative predictions, as the small crack phase often constitutes a substantial portion of the life of many engineering components.

Successful implementation of load spectrum enhancement relies on accurately establishing the relationship between the accelerated factor/compression ratio and the LEF, which in turn requires precise prediction of fatigue life under VA loading. Accurate fatigue life prediction under VA loading must address two challenges: first, the small crack problem. The starting point for calculating structural fatigue life is often a small crack, and the traditional crack growth methodologies based on LEFM tend to yield non-conservative life estimations due to the small crack effect [13,14,15].

Though many scholars have investigated the small crack growth behavior of various materials and proposed several small crack growth models [16,17,18,19,20,21,22], the small crack problem has not been fully resolved. To avoid the complex small crack problem, some scholars [23,24,25,26,27] have introduced the concept of equivalent initial flaw size (EIFS). The core idea is to determine a crack length such that the fatigue life calculated using the long crack growth model aligns with the experimental life data. Liu and Mahadevan [24] proposed that the EIFS can be obtained from the Kitagawa–Takahashi diagram [28]. Zhang et al. [26,27] developed a fatigue life prediction model under CA loading based on EIFS and crack closure, demonstrating good agreement with experimental data for various materials and structures.

The second major challenge is the load interaction effect. Under VA loading, overloads/underloads can induce crack growth retardation/acceleration, significantly influencing the whole fatigue life [29,30,31,32]. The load interaction is usually explained by the crack closure mechanism [33,34,35]. Thus, tracing the variation in crack closure is an effective means to characterize the load interaction effect. In our previous work, Zhang and Liu [36] proposed a virtual crack annealing (VCA) model to compute the crack closure level under CA loading. The VCA model conceptualizes fatigue crack closure zones as annealed material, enabling analytical prediction of crack opening stress through residual stress reversal in an equivalent crack. Further, Jiang et al. [37,38] extended the VCA model to the VA loading conditions by incorporating load interaction effects through variations in crack closure, determined by tracking evolution in the crack tip forward and reverse plastic zones. This modified approach, termed the equivalent plastic zone (EPZ) model, has been validated against crack growth data from VA loading experiments, confirming its effectiveness in characterizing crack tip physical states.

Building on directly upon the foundational concepts of EIFS [24,26,27] and the mechanistic crack closure prediction capabilities of the EPZ model [37,38], this study proposes a VA fatigue life prediction model that explicitly incorporates both the small crack effect and load interaction effect. And it is applied to fatigue load spectrum enhancement. The rest of this paper is organized as follows: First, the material properties, specimens, and experimental setup in this work are introduced. Next, the VA fatigue life prediction model is developed based on the EIFS and EPZ theory. Subsequently, the fatigue life tests of two typical structures under programmed load spectra at different LEFs are conducted to validate the proposed model. Finally, some discussion and conclusions are given.

## 2. Materials

### 2.1. Material Property

The material studied in this work was 2A14 aluminum alloy, with its chemical composition provided in Table 1. The Young’s modulus is 74.7 GPa, and the yield strength and ultimate strength are 377 and 458 MPa, respectively.

**Figure 1:**
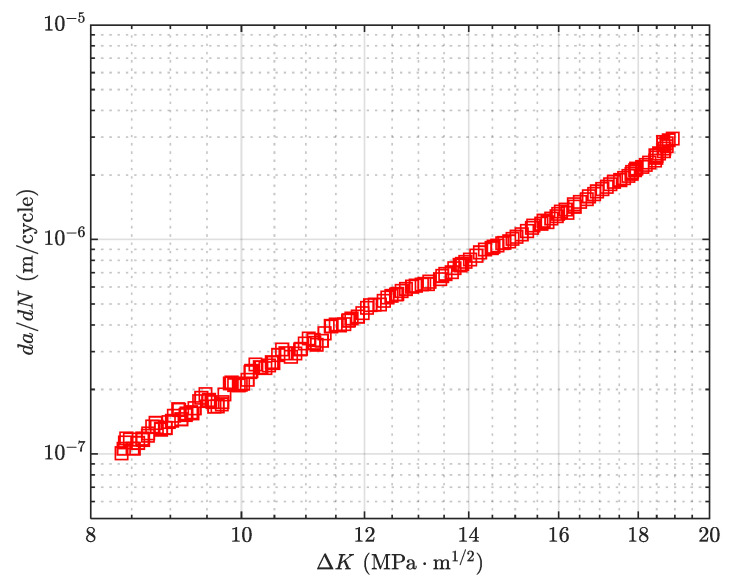
Crack propagation behavior of 2A14 aluminum alloy (*R* = 0.1).

Referring to ASTM E647 [39], the compact tension (CT) specimen was designed to obtain the fatigue crack growth behavior, as illustrated in Figure 1.

### 2.2. Specimen

**Figure 2:**
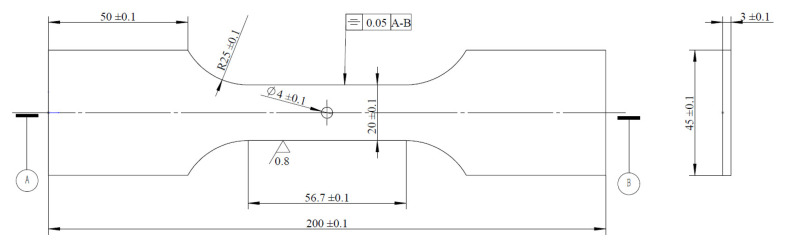
Central-hole specimen (unit: mm).

**Figure 3:**
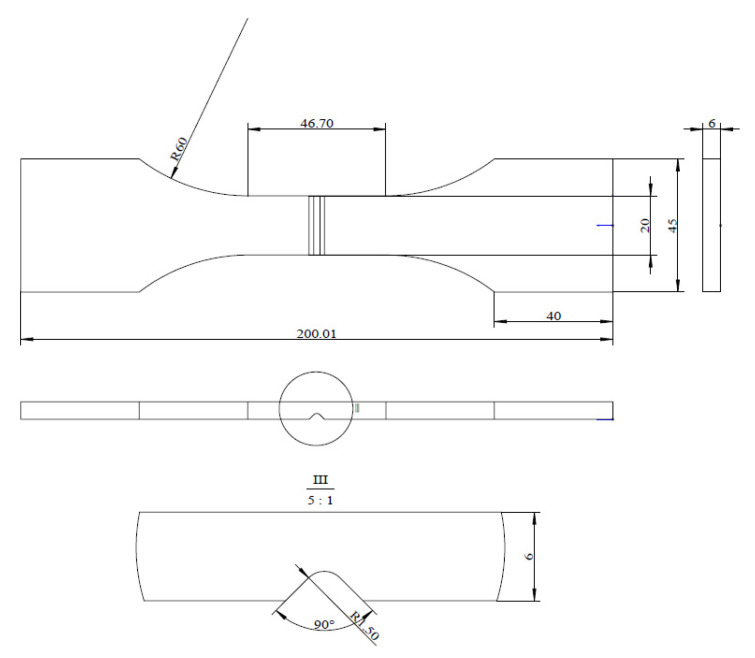
V-notched specimen (unit: mm).

The specimens used in this study include a central-hole specimen and V-notched specimen, with their geometric dimensions shown in Figure 2 and Figure 3, respectively.

### 2.3. Experimental Setup

**Figure 4:**
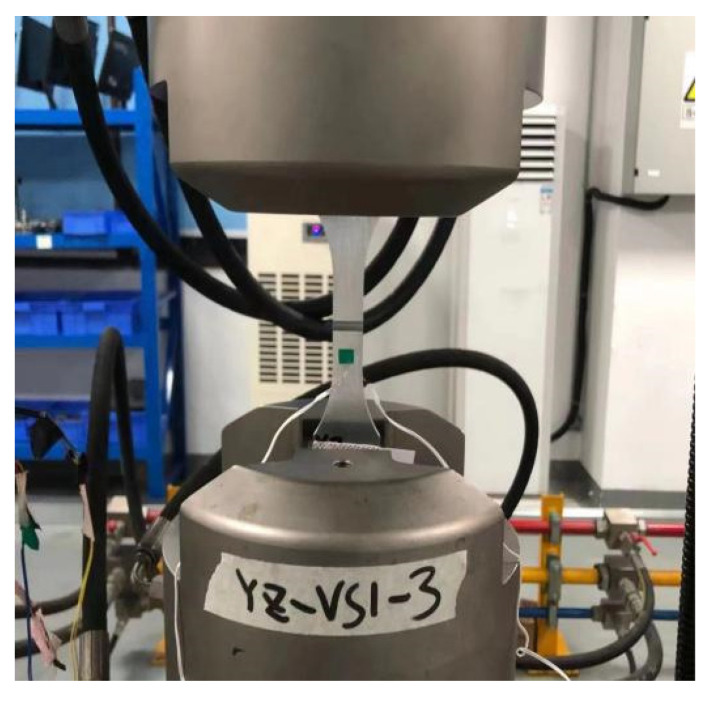
Experimental setup.

All fatigue tests were performed using an MTS Landmark 370.02 hydraulic servo fatigue testing system (MTS Systems Corporation, Eden Prairie, MN, USA, Figure 4) operating at a frequency of 10 Hz under load control.

**Figure 5:**
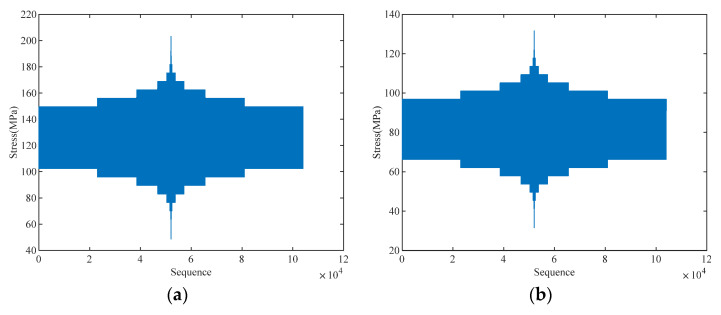
Original load spectra: (**a**) CCTS0; (**b**) VS0.

The original load spectra are 10-level programmed load spectra, denoted as CCTS0 and VS0, respectively, as shown in Figure 5.

Based on the above original spectra, the load spectra were proportionally amplified with LEFs of 1.1 and 1.2. The corresponding enhanced spectra are denoted as CCTS1, CCTS2, VS1, and VS2.

### 2.4. Model Development

#### 2.4.1. Equivalent Initial Flaw Size

**Figure 6:**
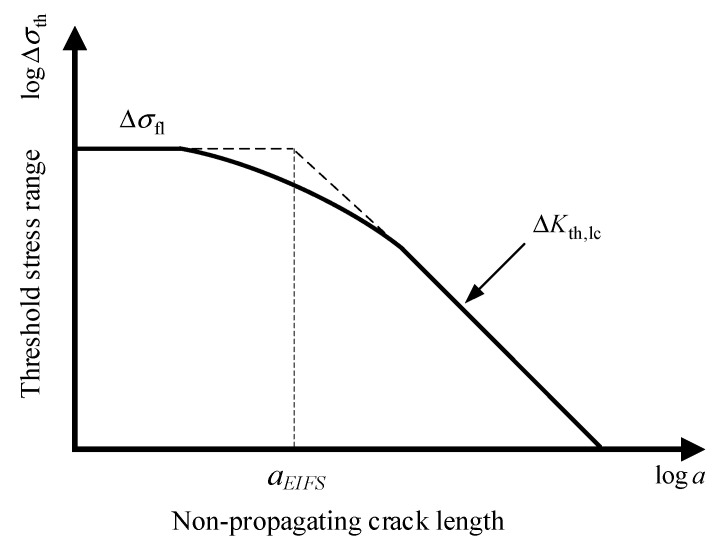
Kitagawa–Takahashi diagram.

As mentioned earlier, some researchers [9,10,11] have introduced the concept of EIFS to avoid the complex small crack problem. Liu and Mahadevan [24] proposed that the EIFS can be calculated from the Kitagawa–Takahashi diagram [28], as illustrated in Figure 6.

The EIFS corresponds to the intersection point of the plain fatigue strength line and long crack threshold stress intensity factor (SIF) line, as expressed as follows:

(1) $$\Delta K_{\mathrm{th},\mathrm{eff}}=\Delta \sigma_{\mathrm{fl}}\sqrt{\pi a_{\mathrm{EIFS}}}YU$$

where a_EIFS_ is the EIFS, ΔK_th,eff_ is the effective threshold SIF range for long cracks, Δσ_fl_ is the plain fatigue strength, *Y* is the geometry correction factor, and *U* is the crack closure ratio.

#### 2.4.2. Equivalent Plastic Zone

**Figure 7:**
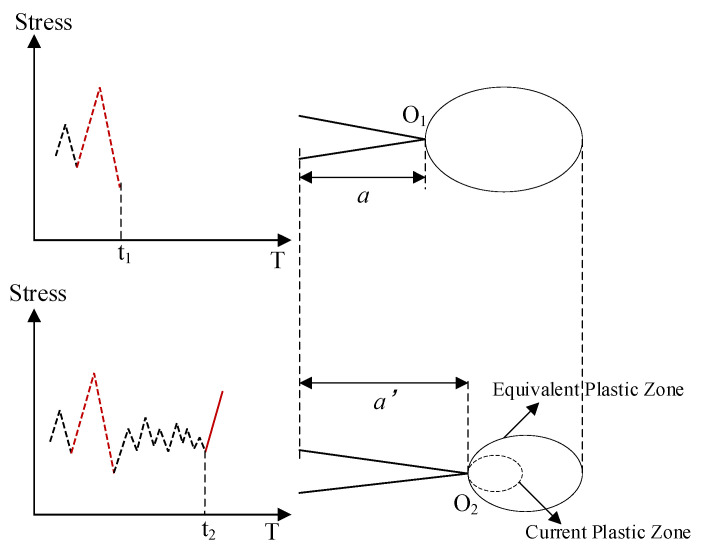
Schematic diagram of equivalent plastic zone.

During cyclic loading and unloading, forward and reverse plastic zones develop periodically at the crack tip. The interaction between these zones reflects the load interaction effect. Therefore, tracing the plastic state at the crack tip provides a means to quantify the influence of load history on fatigue crack growth behavior. Jiang et al. [37] proposed the concept of EPZ to account for the impact of load sequence by tracking the cumulative plastic state resulting from historical loads, as depicted in Figure 7.

Considering an overload occurring at time t1, a large plastic zone is formed at the crack tip (located at point O_1_). Then, the crack propagates within this previously formed large plastic zone. At a later time t_2_, the crack tip moves to point O_2_. If the plastic zone generated by the current load does not exceed the boundary of the historical plastic zone, the EPZ influences the current crack growth behavior rather than the current plastic zone. The size of the EPZ is calculated using Dugdale’s model, as follows:

(2) $$a_{0}+\sum_{j=1}^{i}da_{j}+D_{\mathrm{eq}.i}=\max\left\{a_{0}+\sum_{j=1}^{i}da_{j}+d_{i},\;a_{0}+\sum_{j=1}^{i-1}da_{j}+D_{\mathrm{eq},i-1}\right\}$$

where *D*_eq,i_ is the diameter of EPZ at the i-th cycle, *d*_i_ is the actual plastic zone size at the i-th cycle, *a*_0_ is the initial crack length, and *da_j_* is the crack increment.

Specifically, the EPZ includes the equivalent monotonic and reverse plastic zone:

(3) $$\left\{\begin{array}{l} a_{0}+\overset{i}{\underset{j=1}{\sum }}da_{j}+D_{m,\mathrm{eq},i}=\max\left(a_{0}+\overset{i}{\underset{j=1}{\sum }}da_{j}+d_{m,i},a_{0}+\overset{i-1}{\underset{j=1}{\sum }}da_{j}+D_{m,\mathrm{eq},i-1}\right)\\ a_{0}+\overset{i}{\underset{j=1}{\sum }}da_{j}+D_{r,\mathrm{eq},i}=\max\left(a_{0}+\overset{i}{\underset{j=1}{\sum }}da_{j}+d_{r,i},a_{0}+\overset{i-1}{\underset{j=1}{\sum }}da_{j}+D_{r,\mathrm{eq},i-1}\right) \end{array}\right.$$

where *D*_m,eq,*i*_ and *D*_r,eq,*i*_ are the equivalent monotonic and cyclic plastic zones at the i-th cycle, respectively, and *d*_m,*i*_ and *d*_r,*i*_ are the monotonic and cyclic plastic zones at the i-th cycle, respectively.

The sizes of the monotonic and reversed plastic zones are computed using a modified Dugdale model [40]:

(4) $$\left\{\begin{array}{l} d_{m}=\frac{\pi }{8}{\left(\frac{K_{\max}}{\sigma_{y}}\right)}^{2}\\ d_{r}=\frac{\pi }{8}{\left(\frac{K_{\max}-K_{\mathrm{op}}}{2\sigma_{y}}\right)}^{2} \end{array}\right.$$

(5) $$K_{max}=\sigma_{max}\sqrt{\pi a}Y$$

where *K*_max_ is the peak SIF, *K*_op_ is the crack opening SIF, σ_y_ is the yield stress, σ_max_ is the max stress, and *a* is the crack length.

The EPZ effectively captures the plastic effect caused by load history. Since plasticity is a primary mechanism of crack closure, the influence of the crack-tip plastic state can be described through variations in the crack closure level. Based on the EPZ theory, Jiang et al. [37] derived an analytical solution for the crack closure level under VA loading:

(6) $$\sigma_{\mathrm{op}}=\sigma_{\min,\mathrm{eq}}+\sqrt{{\left(\frac{\sqrt{d_{m,\mathrm{eq}}\frac{8}{\pi^{2}aY}}\sigma_{y}-\sigma_{\min}}{2}\right)}^{2}-\frac{8}{\pi^{2}aY}\sigma_{y}^{2}d_{r}}$$

where σ_op_ is the crack closure level, σ_min,eq_ is the equivalent minimum stress, σ_min_ is the minimum stress, *d*_m,eq_ is the equivalent monotonic crack tip plastic zone size, d_r_ is the reversed crack tip plastic zone size.

#### 2.4.3. Fatigue Life Prediction Model Under Variable Amplitude Loading

**Figure 8:**
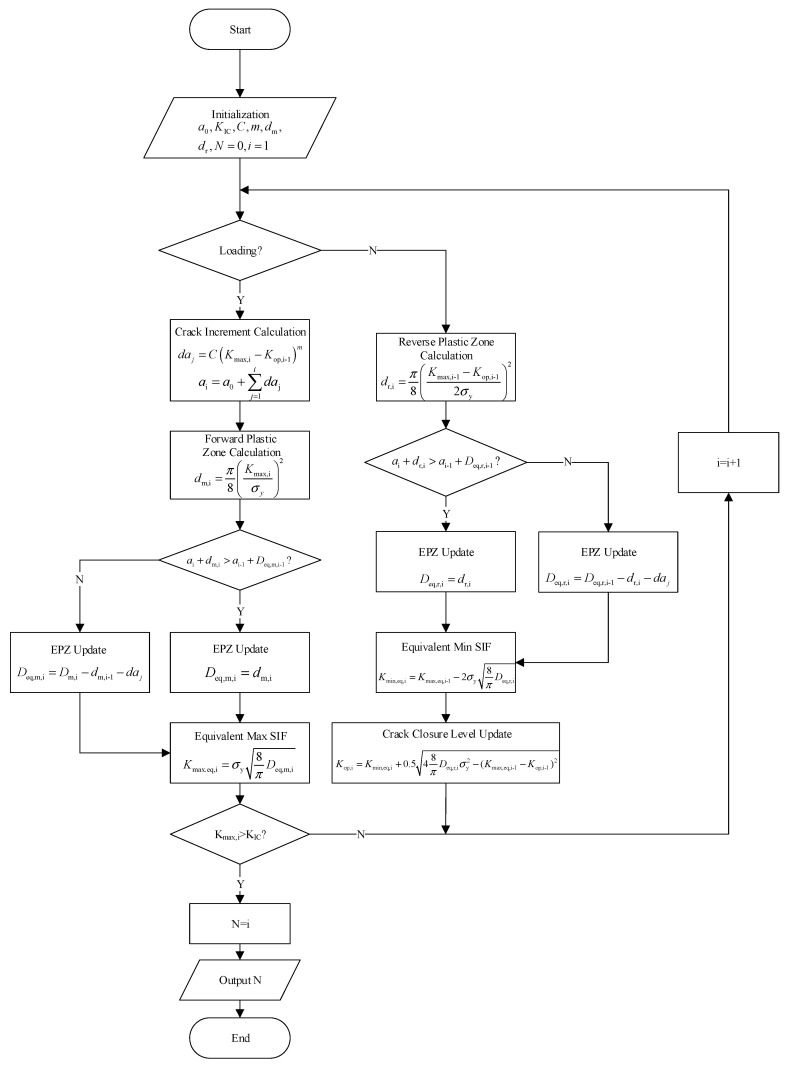
Flowchart of the fatigue life prediction algorithm.

Integrating the EIFS and EPZ theory, the fatigue life prediction process under VA loading is schematically illustrated in Figure 8. It comprises the following steps:

**1: Model initialization.**

Before model prediction, model parameters are calibrated using fundamental material properties (yield strength, fracture toughness) and fatigue crack growth data (*da/dN* versus Δ*K*).

**2: Cycle-by-cycle iteration.**

The VA load spectrum is processed cycle by cycle.

Loading phase: For each cycle, the monotonic EPZ is updated. The corresponding equivalent maximum SIF (*K*_max,eq_) and crack increment (Δ*a*) are calculated.

Unloading phase: The reverse EPZ is updated. The equivalent minimum SIF is determined to estimate the crack closure level for the next cycle.

**3: Termination criterion**

The above process is repeated until the peak SIF exceeds the fracture toughness. The number of cycles consumed in this period is taken as the fatigue life.

## 3. Results and Discussion

### 3.1. Model Prediction

#### 3.1.1. Model Calibration

In this investigation, the Elber model [41] is used to depict the fatigue crack growth behavior, expressed as follows:

(7) $$\frac{da}{dN}=C{\left(\Delta K_{\mathrm{eff}}\right)}^{m}=C{\left(U\Delta K\right)}^{m}$$

where *da/dN* is the crack growth rate, C, m are the model constants, Δ*K*_eff_ is the effective SIF range, and ΔK is the SIF range.

*U* can be obtained by Newman’s empirical formula [42]:

(8) $$U=\frac{1-f}{1-R}$$

(9) $$f=\left\{\begin{array}{ll} \max(R,A_{0}+A_{1}R+A_{2}R^{2}+A_{3}R^{3}) & 0⩽R<1\\ A_{0}+A_{1}R & -1⩽R<0 \end{array}\right.$$

where

$$A_{0}=\left(0.825-0.34\alpha +0.05\alpha^{2}\right){\left[\cos\left(\frac{\pi }{2}\frac{S_{\max}}{\sigma_{0}}\right)\right]}^{1/\alpha }$$

$$A_{1}=\left(0.415-0.071\alpha \right)S_{\max}/\sigma_{0}$$

$$A_{2}=1-A_{0}-A_{1}-A_{3}$$

$$A_{3}=2A_{0}+A_{1}-1$$

The value of *S*_max_/σ_0_ is set to 0.3, consistent with the value for aluminum alloys in NASGRO dataset. α is the constraint factor and related to the stress state. In this study, it is taken as 2. *R* is the stress ratio.

**Figure 9:**
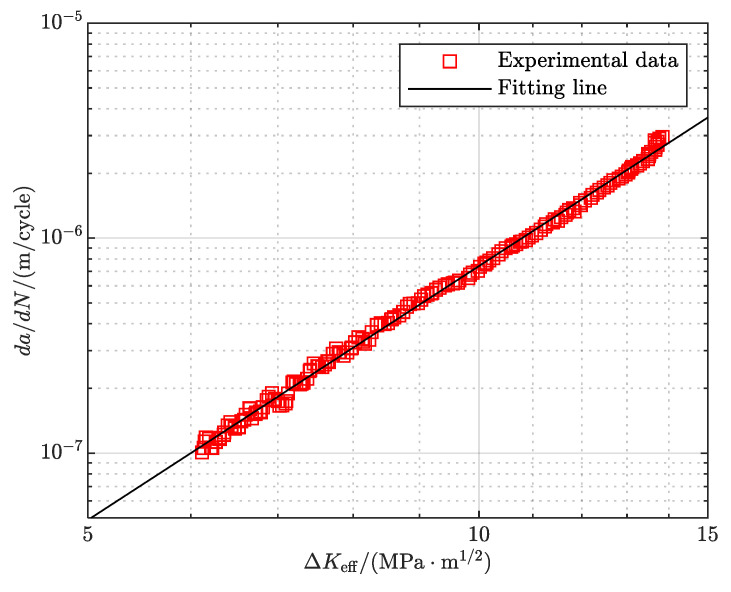
*da/dN* versus Δ*K*_eff_.

Thus, the relationship between crack growth rate and effective SIF range can be obtained as shown in Figure 9. The data exhibit a linear relationship in the log–log coordinate system, confirming the applicability of the Elber model. The material constants *C* and *m* are determined via least-squares fitting, as summarized in Table 2.

#### 3.1.2. Calculation of EIFS

**Figure 10:**
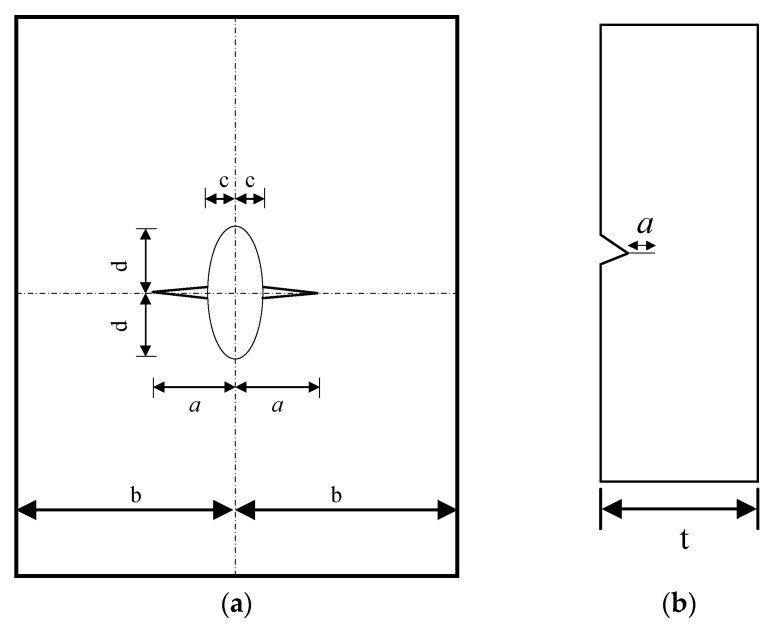
Schematic diagram of crack geometries for typical structures: (**a**) central hole specimen; (**b**) V-notched specimen.

In this study, it is assumed that the crack in the central-hole specimen is an edge-through crack emanating from the central hole, while the crack in the V-notched specimen is treated as a single edge-through crack, as depicted in Figure 10.

The geometry correction factor for an edge-through crack at the central hole is from reference [43]:

(10) $$Y_{\mathrm{CCT}}=\varphi \Psi$$

(11) $$\varphi =\frac{\pi \sqrt{\frac{\tan\bar{\alpha }+g\sin2\bar{\alpha }}{\bar{\alpha }}}\left(1+\frac{\varepsilon^{2}(2-\varepsilon^{2})}{1-\varepsilon }\right)-\sqrt{1+2g}}{\pi -1}$$

(12) $$\psi =h\cdot (3\cdot \beta^{2/3p}-2\cdot \sqrt{h}\beta^{p})$$

where

$$g(\delta )=0.13{\left(\frac{2}{\pi }\arctan\delta \right)}^{2}$$

$$\varepsilon (\delta ,\alpha )=\frac{2}{\pi }\alpha \mathrm{atan}(0.6^{3}\sqrt{\delta })$$

$$p(\delta ,\gamma )=\mathrm{lg}(h^{-3/2})/\mathrm{lg}\beta^{*}$$

$$\beta^{*}(\delta ,\gamma )=\frac{\gamma \delta }{\gamma (2\delta -1)+1}$$

$$h(\delta )=1+\frac{2}{\pi }\mathrm{atan}(1.5\sqrt{\delta })$$

$$a=r+\Delta a,\alpha =a/b,\bar{\alpha }=\alpha \pi /2,\delta =1,\gamma =c/b,\beta =(\alpha -\gamma )/(1-\gamma )$$

For a V-notched specimen, the geometry correction factor is derived from reference [44]:

(13) $$Y_{V}=K_{t}\left[1.12-0.23\left(\frac{a}{T}\right)+10.6{\left(\frac{a}{T}\right)}^{2}-21.7{\left(\frac{a}{T}\right)}^{3}+30.4{\left(\frac{a}{T}\right)}^{4}\right]$$

where *K*_t_ is the theoretical stress concentration factor and *T* is the specimen thickness.

Using Equation (1), the corresponding EIFSs are calculated and presented in Table 3.

#### 3.1.3. Estimation of Crack Closure

**Figure 11:**
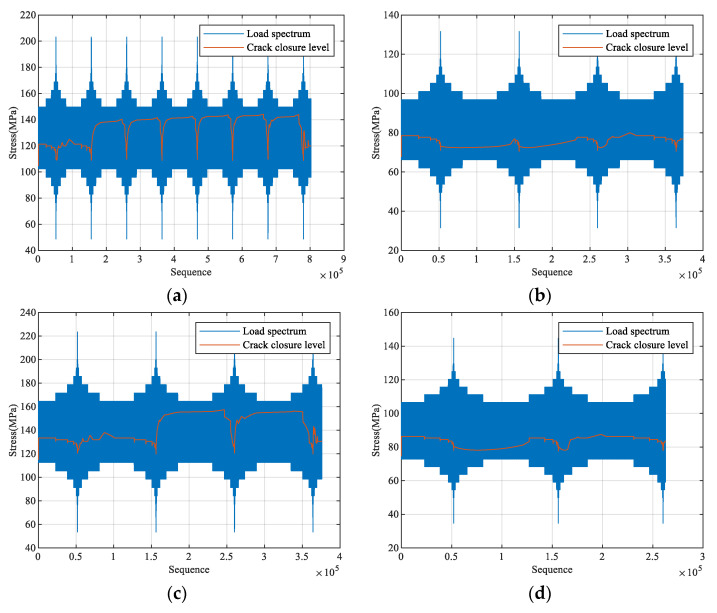

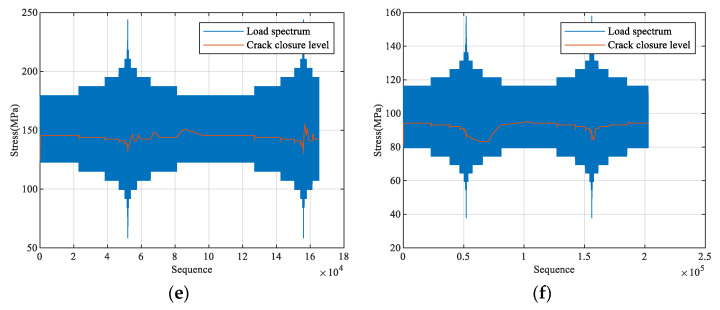
Predicted crack closure level for each enhanced spectrum: (**a**) CCTS0; (**b**) VS0; (**c**) CCTS1; (**d**) VS1; (**e**) CCTS2; (**f**) VS2.

Based on the EPZ theory, the evolution of crack closure level under each enhanced load spectrum is predicted, as shown in Figure 11.

#### 3.1.4. Fatigue Life Prediction

**Figure 12:**
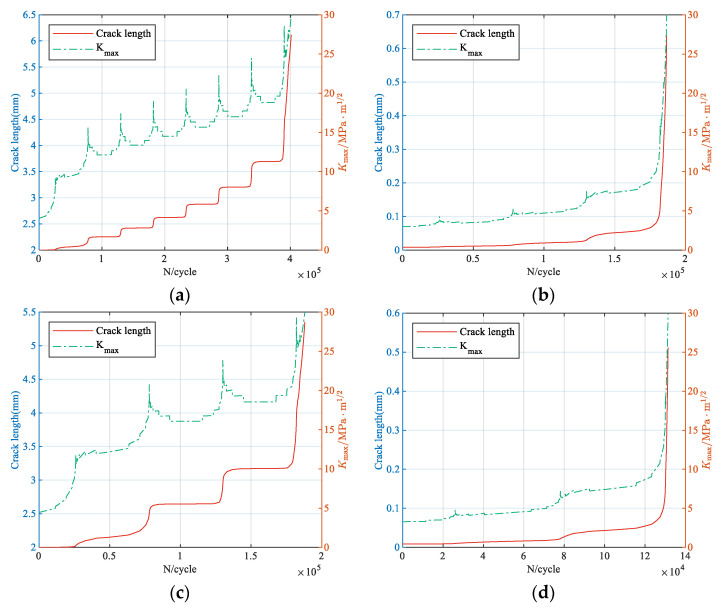

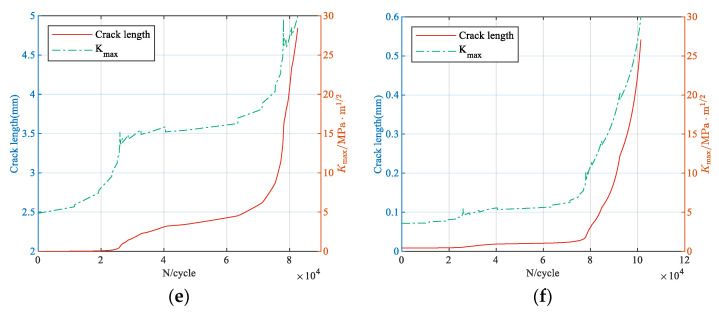
Predicted crack length and *K*_max_ for each enhanced spectrum: (**a**) CCTS0; (**b**) VS0; (**c**) CCTS1; (**d**) VS1; (**e**) CCTS2; (**f**) VS2.

Subsequently, the crack increment is calculated cycle by cycle and accumulated to obtain the *a*–*N* curve. The variations in crack length and peak SIF with the number of cycles for each enhanced spectrum are shown in Figure 12. It can be seen that the peak SIF gradually increases as the crack length increases. Failure is considered to occur when the peak SIF exceeds the fracture toughness. The corresponding cycle count is the fatigue life, as listed in Table 4.

### 3.2. Model Validation

Fatigue life tests were conducted on both central-hole and V-notched specimens under the enhanced spectra, with six parallel specimens tested for each condition. The experimental results, summarized in Table 5 along with their corresponding statistical values in Table 6, reveal a clear trend: fatigue life exhibits a significant decrease with increasing load enhancement factor (LEF).

**Figure 13:**
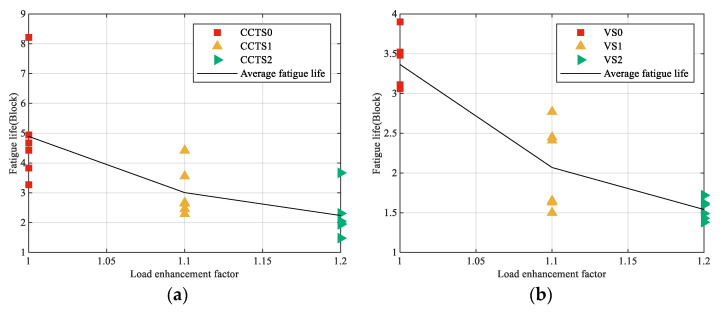
Relationship between fatigue life and LEF: (**a**) central-hole specimen; (**b**) V-notched specimen.

The relationship between fatigue life and LEF for each enhanced spectrum is shown in Figure 13. It is clear that the fatigue life decreases significantly with increasing LEF, which aligns with the fundamental principle that elevated stress amplitudes accelerate damage accumulation [45]. Notably, the application of an LEF of 1.2 resulted in a reduction in the total test time to approximately 50% of that required under the original spectrum, underscoring the practical efficiency of the load enhancement methodology.

**Figure 14:**
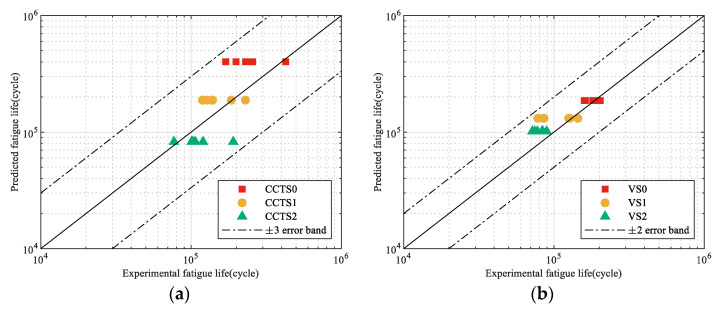
Comparison of model prediction and experimental data under each enhanced spectrum: (**a**) CH specimen; (**b**) V-notched specimen.

A comparison of the model prediction and experimental data for both specimens is presented in Figure 14. For the central-hole specimen, all experimental data fall within a scatter band of three relative to the model prediction. The prediction accuracy is notably higher for the V-notched specimen, with all results confined within a scatter factor of two. This discrepancy in accuracy can be attributed to the differences in crack initiation and early growth behavior between the two specimen geometries. The stress field at the V-notch is more constrained, leading to a more predictable crack initiation site and a reduced small-crack growth phase, which is inherently more stochastic. Generally, the proposed model accurately predicts the fatigue life of the two structures under spectrum loading.

The relative errors of compression ratio for both structures are quantified in Table 7. The central-hole specimen exhibits larger errors than the V-notched specimen, which is consistent with the above observations. The maximum error is about 50%, which remains within an acceptable limit for engineering application [8].

## 4. Conclusions

This study establishes a novel accelerated fatigue testing methodology based on fracture mechanics to investigate fatigue load spectrum enhancement. A new fatigue life prediction model for variable amplitude loading is developed to accurately forecast both the fatigue life and the test time compression ratio. Based on the comprehensive experimental and modeling framework, the key conclusions are as follows:

(1) A fatigue life prediction model for variable amplitude loading is proposed by integrating the theories of Equivalent Initial Flaw Size (EIFS) and the Equivalent Plastic Zone (EPZ). This model provides a physically grounded methodology for load enhancement.

(2) The model prediction is in good agreement with the experimental data. All experimental data fall within a factor of three compared to the model prediction. The maximum error between mean fatigue life and the model prediction is about 50%.

(3) A significant reduction in fatigue testing time is achieved with increasing LEF. Specifically, employing a load enhancement factor (LEF) of 1.2 achieves savings of over 50% in total fatigue testing time.

In the future, the current work will be extended to more loading scenarios.

## Figures and Tables

**Table 1:** Chemical composition of 2A14-T6 aluminum alloy(wt.%).

Element	Si	Fe	Cu	Mn	Mg	Zn	Ti	Al
Min	0.60	0.00	3.90	0.40	0.40	0.0	0.00	Bal.
Max	1.20	0.30	4.80	1.00	0.80	0.3	0.15	Bal.

**Table 2:** Elber model fitting results.

Material	lgC	m	R^2^	RMSE
2A14–T6	−10.05	3.92	0.9977	0.0211

**Table 3:** Estimated EIFS.

Specimen	*K*_t_	Δ*K*_th,eff_/MPa∙m^1/2^	Δσ_fl_/Mpa	*a*_EIFS_/μm
Central hole	3.15	1.03	55	2040
V-notched	5.34	1.03	48	38.7

**Table 4:** Predicted fatigue life for each load spectrum.

	Load Spectrum	Cycle (One Block)	Fatigue Life/Block	Fatigue Life/Cycles	Compression Ratio
Central-hole	CCTS0	52,018	7.71	401,318	0
	CCTS1		3.61	188,011	53.2
	CCTS2		1.59	82,608	79.4
V-notched	VS0	52,026	3.59	186,718	0
	VS1		2.53	131,367	29.5
	VS2		1.95	101,403	45.7

**Table 5:** Fatigue life for each load spectrum.

	Load Spectrum	Cycles	Fatigue Life/Block	Fatigue Life/Cycles
Central-hole	CCTS0	52,018	3.27 4.67 4.94 3.83 8.21 4.43	170,540 242,924 256,969 199,229 427,067 230,440
	CCTS1		2.67 2.46 2.64 2.29 4.42 3.56	138,889 127,964 137,328 119,121 229,920 184,945
	CCTS2		2.05 1.98 3.67 1.48 1.94 2.31	106,637 102,996 190,906 76,987 100,915 120,162
V-notched	VS0	52,026	3.52 3.11 3.06 3.11 3.48 3.90	183,131 161,801 159,200 161,801 181,051 202,901
	VS1		2.77 1.63 1.65 1.50 2.45 2.41	144,112 84,802 85,843 78,039 127,464 125,383
	VS2		1.60 1.38 1.62 1.43 1.72 1.49	83,242 71,796 84,282 74,397 89,485 77,519

**Table 6:** Mean fatigue life and compression ratio for each load spectrum.

Specimen	Load Spectrum	Cycles	Mean Life /Block	Fatigue Life /Cycles	Compression Ratio/%
Central-hole	CCTS0	52,018	4.89	254,368	0
	CCTS1		3.00	156,054	38.7
	CCTS2		2.24	116,520	54
V-notched	VS0	52,026	3.36	174,807	0
	VS1		2.07	107,694	37.27
	VS2		1.54	82,013	52.23

**Table 7:** Comparison of predicted and experimental compression ratio.

Specimen	Load Spectrum	Experimental Value/%	Predicted Value/%	Error/%
Central-hole	CCTS0	0	0	0
	CCTS1	38.7	53.2	52.2
	CCTS2	54	79.4	47.0
V-notched	VS0	0	0	0
	VS1	37.27	29.5	−20.8
	VS2	52.23	45.7	−12.5

## Data Availability

The data presented in this study are available on request from the corresponding author due to privacy.

## Abbreviations

The following abbreviations are used in this manuscript:

CA	Constant amplitude
EIFS	Equivalent initial flaw size
EPZ	Equivalent plastic zone
LEF	Load enhancement factor
LEFM	Linear elastic fracture mechanics
SIF	Stress intensity factor
VCA	Virtual crack annealing
VA	Variable amplitude

## Nomenclature

**Symbol**	**Illustration**
*a*	Crack length
*a*_0_	Initial crack length
*a*_EIFS_	Equivalent initial flaw size
A_i_(i = 1,2,3)	Crack closure coefficients
C,m	Material constants
*da*	Crack increment
*da/dN*	Crack growth rate
*d*_m_	Monotonic crack tip plasticity zone size
*d*_r_	Reverse crack tip plasticity zone size
*D*_eq_	Equivalent crack tip plasticity zone size
*D*_m,eq_	Monotonic equivalent crack tip plasticity zone size
*D*_r,eq_	Reverse equivalent crack tip plasticity zone size
*f*	Crack closure parameter
*K*_t_	Theoretical stress concentration factor
*K*_max_	Maximum stress intensity factor
*K*_op_	Crack opening stress intensity factor
Δ*K*	Stress intensity factor
Δ*K*_eff_	Effective stress intensity factor
Δ*K*_th_	Threshold stress intensity factor
Δ*K*_th,eff_	Effective threshold stress intensity factor
*R*	Stress ratio
*T*	Specimen thickness
*U*	Crack closure ratio
*Y*	Geometric correction factor
σ_max_	Maximum stress
σ_min_	Minimum stress
σ_min,eq_	Equivalent minimum stress
σ_op_	Crack closure stress
σ_y_	Yield strength
Δσ_fl_	Fatigue limit
